# RAGMail: a cloud-based retrieval-augmented framework for reducing hallucinations in LLM text generation

**DOI:** 10.1038/s41598-026-38913-w

**Published:** 2026-02-09

**Authors:** Priyodip Sanyal, Kumud Rathore, R. Vijaya Arjunan

**Affiliations:** Manipal Institute of Technology, Manipal Academy of Higher Education, Manipal, India

**Keywords:** AI personalization, Cold email generation, Cloud native architecture, Large language models, Factualness evaluation, Weighting LLM, Natural language processing, Generative AI, Engineering, Mathematics and computing

## Abstract

Cold emailing is used to personalize, target emails for outreach without prior contact. Automating this personalized cold email generation process can significantly improve outreach efficiency for job seekers, particularly in competitive industries. It streamlines the process of composition, saves time and increases engagement, tailored to a specific industry or role. In today’s competitive market, where job application is made easy, such a tool scales communication and boosts the conversion rate. The cold email generator. RAGMail, is an intelligent cold email generator that is cloud-integrated and uses Retrieval-Augmented Generation (RAG) to reduce hallucinations. The cloud-native infrastructure on which the system is built makes use of services including managed Large Language Model (LLMs) APIs, scalable vector databases, and object storage. With real-time document retrieval and cloud-hosted, metadata-aware templates, RAGMail guarantees high personalization accuracy and factual foundation. This cloud-native architecture provides elastic scalability, low-latency inference, and real-time personalization at scale, all while protecting data and user privacy with role-based access control and encrypted storage. Beyond job applications, the approach can be applied to a wide range of outreach sectors, including sales, academia, and commercial relationships, where factual accuracy and context sensitivity are critical. The system ensures high availability and load balancing during peak demand periods by utilizing distributed cloud resources. The models exhibit open-domain conversational capabilities, generalize effectively to scenarios beyond the trained data, and as verified by human evaluations, substantially reduce the well-known problem of knowledge hallucination in state-of-the-art chatbots. The proposed framework offers a scalable and reliable solution for generating contextually grounded, high-quality cold emails using Retrieval-Augmented Generation.

## Introduction

Cold emailing serves as a critical strategy for candidates seeking to enhance their visibility to recruiters in a competitive job market. Crafting concise, well-structured messages that present tailored value propositions allows candidates to differentiate themselves effectively. Personalized emails enable applicants to demonstrate genuine interest and alignment with the recruiter’s needs, fostering a sense of empathy and relevance. Such targeted communication increases the likelihood of engagement by making the recipient feel acknowledged in their specific context and professional challenges^1,2^. This tailored strategy is essential for engaging recipients, promoting interaction, and ultimately facilitating desired outcomes like conversion^2^.

Personalization is a fundamental aspect of automated email generation, serving both functional and strategic purposes. It enhances the uniqueness of each message, thereby reducing the likelihood of classification as spam, while simultaneously signalling to the recipient that the content is specifically tailored to their needs. Incorporating publicly available personalization elements—such as the recipient’s role, company, or recent activity—further strengthens the relevance and effectiveness of the out^3^.

Cold emails, when crafted effectively, can open doors to valuable job opportunities. However, writing such emails manually requires significant time and effort and may still lack relevance if the candidate fails to align their message with the specific job requirements.

Large Language Models (LLMs) are sophisticated Artificial Intelligence (AI) systems that can comprehend and produce language that is like that of humans to carry out challenging jobs. They can perform almost human-like tasks like summarizing and translating thanks to advancements in training and architecture. LLMs are effective tools for automating jobs in a variety of industries, including data analysis, customer service, and content generation, thanks to these features^3^.

Emails generated by traditional LLMs often suffer from a common drawback: hallucination. This refers to the generation of incorrect or fabricated information that does not exist in the source data. Such inaccuracies can damage a candidate’s credibility and reduce the effectiveness of their outreach.

To overcome hallucinations in LLM-generated content, the proposed study employs a Retrieval-Augmented Generation (RAG) framework, which combines the strengths of parametric and non-parametric memory^4,5^. This approach enables the system to dynamically retrieve semantically relevant job-related information such as required skills, qualifications, and responsibilities from external sources before generating the cold email. By grounding the generation process in factual and context-rich data, RAG significantly enhances the accuracy and specificity of the output^6^. Unlike conventional LLM pipelines that rely solely on internal training knowledge, this method ensures that the generated emails are closely aligned real-world job postings^7^.

Cloud computing enables on-demand access to shared IT resources like servers, storage, and applications, typically managed by third-party providers. It supports scalable, virtual, and distributed systems, offering cost-efficiency through pay-per-use models. Recognized as a top disruptive technology, major companies like Google and Amazon are investing in global data centers to ensure reliability. Cloud computing is widely adopted for its flexibility and fast deployment, with ongoing research exploring its history, models, characteristics, opportunities, and challenges.

*XaaS* (Anything or Everything as a Service) refers to the broad and evolving category of services delivered over the internet. It encompasses models such as Software as a Service (SaaS), Platform as a Service (PaaS), and Infrastructure as a Service (IaaS), among others. These services offer scalable, on-demand access to computing resources, platforms, applications, storage, and even AI tools, minimizing the need for physical infrastructure and reducing operational costs. XaaS plays a critical role in enabling digital transformation, enhancing agility, and supporting innovation in cloud-based environments^8^.

A recently introduced technique for open-domain question answering is the neural retrieval-in-the-loop method known as Retrieval-Augmented Generation (RAG) which has demonstrated significant effectiveness in generating factually accurate and contextually relevant responses by grounding the output in retrieved external knowledge^9^.

Knowledge hallucination in conversational agents arises irrespective of language or model size or train data. RAG seems like a promising solution as it reduces hallucination by generalizing beyond the training data on unseen distribution^9^.

Nearest neighbor language models are highly dependent on the similarity of context distribution between training and test dataset for interpolating prediction. This approach is effective only for knowledge, names, and near duplicate sentences^10^.

**Fig. 1:**
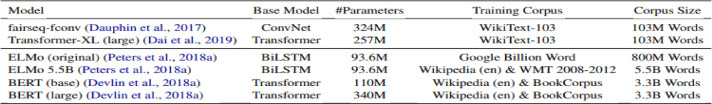
Language models considered in this study.

Figure 1 represents pre-trained neural language models that have demonstrated a remarkable capacity to assimilate a considerable amount of intricate knowledge from datasets. They achieve this without reliance on external memory, instead functioning as a parameterized implicit knowledge repository. Although this advancement is notable, such models come with their limitations: they are unable to easily augment or modify their memory, do not readily elucidate the rationale behind their predictions, and may generate “hallucinations” that could mislead users or disseminate misinformation, thereby raising significant concerns regarding their reliability and safety in critical applications^11^.

A persistent issue in LLMs is hallucination, where models generate plausible but incorrect or fabricated information. Hallucinations can undermine the credibility of AI systems, particularly in high-stakes scenarios. Research classifies hallucinations into intrinsic and extrinsic types. Intrinsic hallucinations occur when the model internally generates inconsistencies, often due to errors in reasoning or misinterpretation of learned patterns. Extrinsic hallucinations, on the other hand, arise when the model produces outputs that are factually incorrect concerning the external world, often due to limitations in the training data or retrieval mechanisms^1^.

A hybrid approach that combines Retrieval-Augmented Generation (RAG) and Factualness Evaluation via Weighting LLMs (FEWL). The core motivation behind this system is to support job seekers in crafting personalized and context-aware outreach messages to recruiters, particularly in competitive hiring environments where a generic or misaligned email may lead to being overlooked^6^.

Cloud computing encompasses both the applications delivered as services over the Internet and the underlying hardware and system software within data centres that enable those services. These services are commonly categorized as Software as a Service (SaaS), while other models include Infrastructure as a Service (IaaS) and Platform as a Service (PaaS). The growing demand for cloud computing is driven by its numerous benefits to individuals, consumers, and businesses, such as cost reduction, simplified access, minimal management overhead, free service offerings, and improved reliability^12^. Cloud-based services are particularly advantageous for businesses with fluctuating or growing bandwidth needs, as they allow easy scalability by utilizing remote server resources.

The proposed system extracts job descriptions from career portals and candidate information from resumes, aligning them to create tailored emails. Unlike traditional text generation methods, our approach integrates FEWL to assess the factual correctness of generated content by assigning weights to different aspects such as skills, experience, and job relevance. By implementing this mechanism, we mitigate the risk of hallucinations in AI-generated emails, ensuring credibility and personalization. Experimental results demonstrate that emails generated with factual evaluation outperform generic AI-generated emails in accuracy and recruiter engagement, thereby improving response rates.

## Related work

Artificial Intelligence Generated Content (AIGC) has become increasingly popular in recent years. Several content creation tools have been meticulously designed to produce diverse outputs^13^. Large language models (LLMs) are transformer-based neural network architectures that have been pre-trained with language models as loss functions using many model parameters and vast amount of test data^14^. LangChain, a modular framework for integrating Large Language Models with diverse data sources, streamlines the development of intelligent applications and accelerates innovation in AI-driven solutions^15^.

Retrieval augmentation is a common method for using a Transformer encoder with a large context, but only some of it is relevant. With this approach, a retrieval system locates and chooses a portion of the context to be included in the final encoding that the decoder handles^12^.

RAG models achieve state-of-the-art outcomes in open-domain quality assurance. People prefer RAG generation over parametric BART due to its factual and specific nature^16^.

Retrieval augmented text generation relies heavily on retrieval quality, which refers to the similarity between the query and retrieved examples^17^. In-Context RALM demonstrates that Retrieval-Augmented Generation using frozen large language models significantly boosts performance in open-domain question answering by enhancing factuality and relevance without additional fine-tuning^18^.

Bidirectional encoding is used in Retrieval-Augmented Generation (RAG) systems to generate semantically rich vector representations of both queries and documents. Models like BERT (Bidirectional Encoder Representations from Transformers) and RoBERTa (Robustly Optimized BERT Approach), which are based on bidirectional Transformers, analyze the full context of a sentence—looking both left and right of each word—allowing for deeper understanding of meaning. In RAG, these encoders embed documents and user queries into dense vectors, which are then compared using similarity measures (e.g., cosine similarity) to retrieve the most relevant context. This improves retrieval accuracy and ensures that the retrieved passages align better with the user’s intent, ultimately enhancing factuality and reducing hallucinations in the generated output^19^.

Efforts to mitigate hallucinations in LLMs focus on several key strategies. First, improving retrieval mechanisms by incorporating high-quality, domain-specific databases helps ensure that relevant and accurate information is supplied to the model. Second, refining prompt engineering techniques, such as explicitly directing the model to verify its sources, has been shown to enhance factual consistency. Additionally, filtering retrieved documents through verification layers can prevent the model from incorporating unreliable information. Studies also suggest the effectiveness of confidence scoring and uncertainty quantification, where models indicate the reliability of their generated responses, allowing users to assess the trustworthiness of outputs^20^. Further, fine-tuning models with human feedback and Reinforcement Learning from Human Feedback (RLHF) has been demonstrated to reduce hallucination rates by aligning generated content with human expectations and factual accuracy^21^.

Future work should focus on adaptive learning approaches that dynamically adjust retrieval strategies based on context and model confidence levels. By improving these components, the reliability of RAG in minimizing hallucinations can be further enhanced, contributing to the broader goal of developing more trustworthy AI-driven applications^22^.

Query-based RAG augments the user query with retrieved information to form a unified prompt for the generator, enhancing relevance and factuality across modalities without additional model training. While large language models (LLMs) may hallucinate, they retain substantial factual knowledge from pretraining, often producing truthful outputs when multiple responses are compared for the same prompt^4,22^.Query-based RAG, often paired with LLM generators, offers modular flexibility, allowing swift integration of pre-trained components for quick deployment. Prompt design is crucial for utilizing retrieved data within this setup^23^. Llama models, trained on public data, achieve competitive performance with state-of-the-art models like GPT-3 and PaLM, demonstrating that open-access training can yield high-performing large language models^24^.

Older GPT versions are less effective in modern RAG setups due to lower contextual capacity, higher hallucination risk, weak instruction alignment, and poor integration support. Newer models not only reduce hallucinations but also enable modular, prompt-driven, and real-time knowledge-enhanced generation, making them ideal for applications like RAGMail or cold email generation.

A low-cost approach to analyzing model behaviour is prompt-based hallucination induction, which produces only slight gains over fine-tuning-based methods. Recent research has shown that, while prompts can elicit fabricated responses for contrastive training, fine-tuned models achieve significantly greater gains in reducing hallucinations^25^.

Self-contradiction, a form of hallucination where large language models generate conflicting statements in the same context, has been shown to affect over 17% of ChatGPT outputs. Recent work introduces a prompt-based black-box framework for detecting and mitigating such contradictions without relying on retrieval. This method complements RAG, as many contradictions cannot be verified through external sources, and achieves high F1 scores in detection while preserving fluency^26^.

Knowledge-graph-driven summarization and integrating Natural Language Processing techniques such as word segmentation, part-of-speech tagging, and Named Entity Recognition (NER) to construct knowledge graphs from Mobile Edge Computing (MEC) text data, RAGMail employs a similar strategy to enhance factual accuracy in cold email generation. In their work, semantic representations obtained via Graph Convolutional Network (GCN) were used to guide a Pointer-Generator Network (PGN) PGN-based summarizer, enabling efficient multi-document summarization directly on edge devices, thereby reducing inference latency and minimizing dependence on centralized servers. Inspired by this, RAGMail introduces a retrieval-augmented email generator grounded in a lightweight domain-specific knowledge graph that captures entities such as organizations, roles, and recent events relevant to the target audience. This hybrid model can generate new text while also preserving exact factual details from the source — which is crucial for avoiding hallucination and maintaining specificity The graph-structured context is encoded and injected into a retrieval-augmented decoder to maintain factual consistency and reduce hallucination, particularly in templated sections like greetings, value propositions, and calls to action. Furthermore, by enabling client-side inference, RAGMail preserves user privacy while delivering rapid and reliable email generation, demonstrating improved factual grounding compared to standard RAG methods^27^.

One promising area of research is fine-tuning LLMs for domain-specific tasks within the RAG framework. LLMs, although powerful, can exhibit biases or knowledge gaps when applied to specific domains This targeted fine-tuning can significantly enhance data extraction accuracy, particularly in specialized applications like e-commerce product scraping. Researchers have proposed similar approaches, demonstrating that tailored LLMs can enhance extraction quality and foster the development of specialized RAG models for web scraping tasks.

Existing research on cloud application cost optimization emphasizes the significance of early-stage, model-driven decision-making for efficient service deployment, particularly from the application provider’s perspective where traditional reactive cost-control methods often fall short. One notable approach proposes a cloud application context metamodel, which encapsulates infrastructure and service-specific parameters that influence both deployment cost and Quality of Service (QoS). By simulating various deployment configurations, this framework enables the selection of optimal strategies that balance cost-efficiency and service reliability^28^.

RAGMail introduces a lightweight pre-deployment modeling layer tailored for retrieval-augmented text generation. Rather than treating generation as agnostic to its runtime context, RAGMail conditions both its retriever and decoder modules on cloud deployment parameters, including infrastructure constraints, SLA-inspired quality thresholds, and resource allocation policies. This deployment-aware conditioning enhances factual alignment and reduces hallucination rates, especially in cost-sensitive use cases like cold outreach automation.

Recent efforts to address hallucinations in LLMs include FActScore^29^ for atomic factuality evaluation, FELM^30^ for holistic factuality benchmarking, and SelfCheckGPT^31^ for hallucination detection without ground-truth references. FactLens^32^ further extends fact verification by enabling fine-grained claim-level analysis. These works collectively demonstrate the emerging consensus on multi-dimensional evaluation of factuality, which directly motivates the FEWL approach proposed in this study.

Recent studies have expanded the landscape of cloud-based AI security and distributed learning. For example, incentive mechanisms in federated learning have been optimized for vehicular networks^33^, while weaknesses in object storage policies have exposed cloud content security risks^34^. Blockchain deception models such as AutoD and its distributed variant DeepAutoD^35^ further highlight the intersection of machine learning and security. Graph neural networks have also been applied for recognizing BGP communities^36^, while techniques like Gradient Shielding^37^ aim to mitigate vulnerabilities in deep models. These works collectively inform our study by situating RAGMail at the intersection of hallucination reduction, cloud reliability, and secure distributed AI.

## Cloud-Aware architecture design

To ensure that the RAGMail system is scalable, efficient, and cost-effective to deploy, we use a cloud-aware architecture approach that takes infrastructure constraints, quality of service (QoS) needs, and service-specific aspects into account throughout the generation pipeline. RAGMail facilitates a dynamic and resource-efficient inference process by incorporating cloud context into both the retriever and decoder stages, in contrast to conventional generative systems that function independently of their deployment environment.

This architecture draws inspiration from cloud application cost optimization frameworks, such as the Cloud Application Context Metamodel proposed by Ciccozzi et al.^38^, which models critical deployment parameters—such as CPU/GPU allocation, latency requirements, and service-level agreement (SLA) constraints. These models have demonstrated effectiveness in reducing operational expenditures while preserving quality of service (QoS), particularly in simulation-based, pre-deployment decision-making scenarios.

To support hallucination-resistant text generation in a cost-effective and latency-aware manner, RAGMail incorporates a lightweight, cloud-aware modeling layer. This layer is designed to condition both the retriever and decoder stages of the Retrieval-Augmented Generation (RAG) pipeline on deployment-specific context, such as infrastructure capacity, quality-of-service (QoS) thresholds, and cost constraints. This approach enables the system to maintain factuality and reliability while optimizing for operational efficiency in a cloud environment.

### A. Cloud Context Metamodel

The architectural design of RAGMail is informed by prior research in context-aware deployment optimization for cloud-based applications. Central to this approach is the implementation of a cloud application context metamodel, which encapsulates infrastructure-level and service-level parameters. These parameters include instance type specifications, latency expectations, throughput limitations, cost models, and deployment region preferences. By capturing such configuration details, the metamodel serves as an abstraction layer that allows the system to simulate and evaluate deployment scenarios prior to execution. This simulation-driven methodology supports more informed orchestration decisions, enabling the system to proactively select configurations that optimize both performance and cost efficiency^39^.

### B. SLA-Aware Parameter Conditioning

Extending the metamodel framework, RAGMail incorporates soft Service-Level Agreement (SLA) constraints into its inference pipeline. This design follows SLA-driven provisioning strategies proposed by Buyya et al. and allows for intelligent adaptation during runtime. The system continuously monitors indicators such as retriever load, vector search latency, and token generation rates. When internal latency estimates indicate potential SLA violations—such as surpassing a target 95th percentile response time of 700 milliseconds—RAGMail automatically modulates the behavior of its generation components. The retriever and decoder modules dynamically adjust their respective processing scopes, either by constraining context windows, shortening retrieved content segments, or reducing token output lengths. These modifications aim to reduce computational latency without compromising the factual quality of generated text, thus ensuring that semantic integrity is maintained within the bounds of service reliability^40^.

### C. Cloud-Aware Retrieval Pipeline

The cloud-aware retrieval pipeline integrates these modeling and SLA-aware mechanisms into a cohesive runtime workflow. Initially, the system performs metamodel instantiation by collecting deployment-specific details such as vector retrieval latency, available memory, instance types, and system throughput capabilities. These runtime parameters populate the cloud context metamodel. Subsequently, the system engages in simulation-based evaluation to estimate whether current configurations and system load are compatible with predefined SLA targets. If the projected performance suggests a risk of SLA non-compliance, the pipeline transitions into adaptive execution. During this phase, the system modifies its retrieval and generation behaviors in real time to align with operational constraints. Such dynamic adaptation prevents excessive resource usage and ensures cost-efficient deployment while upholding the semantic fidelity of generated content. This approach is especially advantageous during cold-start or exploratory inference phases, where computational overhead is typically high and needs to be carefully managed.

**Fig. 2:**
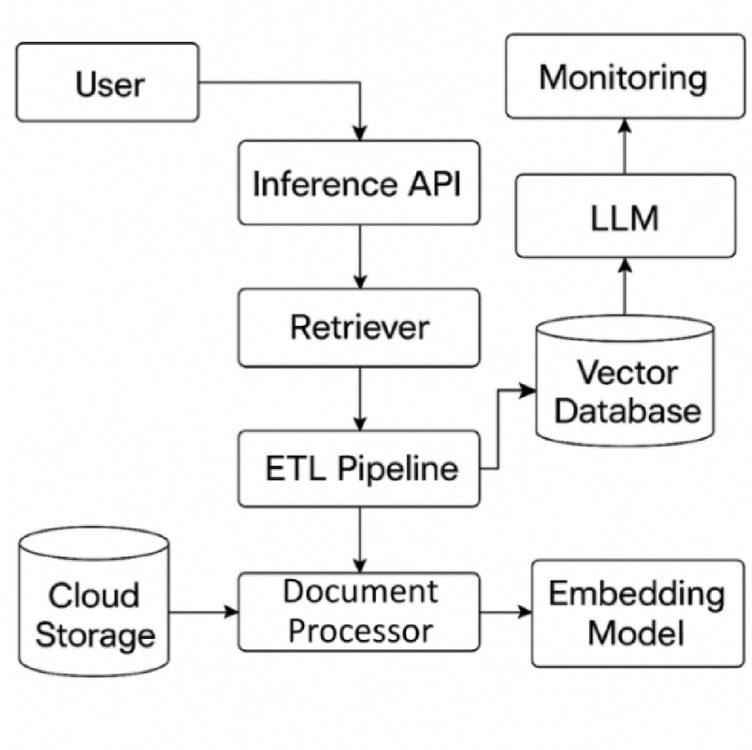
Cloud-Aware Architecture for RAGMail Generation Workflow.

Figure 2 illustrates the cloud-aware architecture of RAGMail, a retrieval-augmented generation system designed to minimize hallucinations in large language model (LLM) outputs. The workflow begins with the user query, which is submitted through the Inference API. This API orchestrates the request, directing it to the retriever component for context fetching.

To enable semantic grounding, relevant documents are pre-processed through an ETL pipeline. Raw documents stored in Cloud Storage are passed to the Text Preprocessor, where they undergo cleaning, segmentation, and metadata enrichment. The cleaned chunks are then embedded into high-dimensional vectors via an Embedding Model, which captures semantic representations.

These vectors are stored in a Vector Database, allowing the retriever to perform similarity search using the user’s embedded query. The top-ranked retrieved chunks are forwarded along with the query to the LLM, which generates contextually grounded responses.

A dedicated Monitoring Layer continuously tracks infrastructure metrics (e.g., latency, throughput, token usage) and semantic metrics (e.g., hallucination rate, factuality score). This observability ensures SLA compliance, supports adaptive throttling, and enables fine-tuning decisions. Tools such as Prometheus, Grafana, and CloudWatch are used for real-time telemetry and dashboard visualization.

#### Cloud-Aware adaptive execution

To ensure SLA compliance, RAGMail implements adaptive execution strategies governed by latency thresholds (e.g., **700 ms at the 95th percentile**). Monitoring is achieved through **Prometheus**, while dashboards in **Grafana** visualize breaches in real time. SLA violations trigger automated actions, including retrieval depth reduction, token output truncation, or temporary fallback to cached responses. These runtime adaptations are coordinated through APIs such as **AWS Auto Scaling** and **Google Cloud Monitoring**, enabling dynamic modulation of resource allocation.

*Pseudocode for SLA feedback loop*:

while service_running:

latency = monitor_prometheus().

if latency > SLA_threshold:

adjust_retriever(depth-1).

shorten_output_tokens().

scale_resources().

Empirically, reducing retrieval depth by one passage resulted in < 3% drop in factual alignment (measured by FActScore and FEWL), indicating acceptable trade-offs between SLA enforcement and semantic fidelity. Figure X illustrates the feedback loop architecture linking monitoring tools, adaptive logic, and retriever-decoder modules.

The architecture is designed to be fully cloud-native, with elastic resource provisioning, containerized components, and support for adaptive autoscaling. This ensures that the system remains cost-efficient, fault-tolerant, and reliable under varying load conditions, particularly during high-volume cold email generation or exploratory inference tasks.

## Method

Cold Email Generator provides an effective, automated solution for targeted outreach using Llama and LangChain. The system can analyze job listings, extract key data, and produce highly relevant content for emails catered to roles and skill sets by utilizing Llama, an effective open-source large language model. For contextual comprehension and real-time processing, LangChain easily links the model with data sources such as vector databases and job websites^41^.

The system integrates key components of modern Natural Language Processing (NLP), including web scraping, resume parsing, information retrieval, and large language models (LLMs), to automate and optimize the process of email generation^23^.

**Fig. 3:**
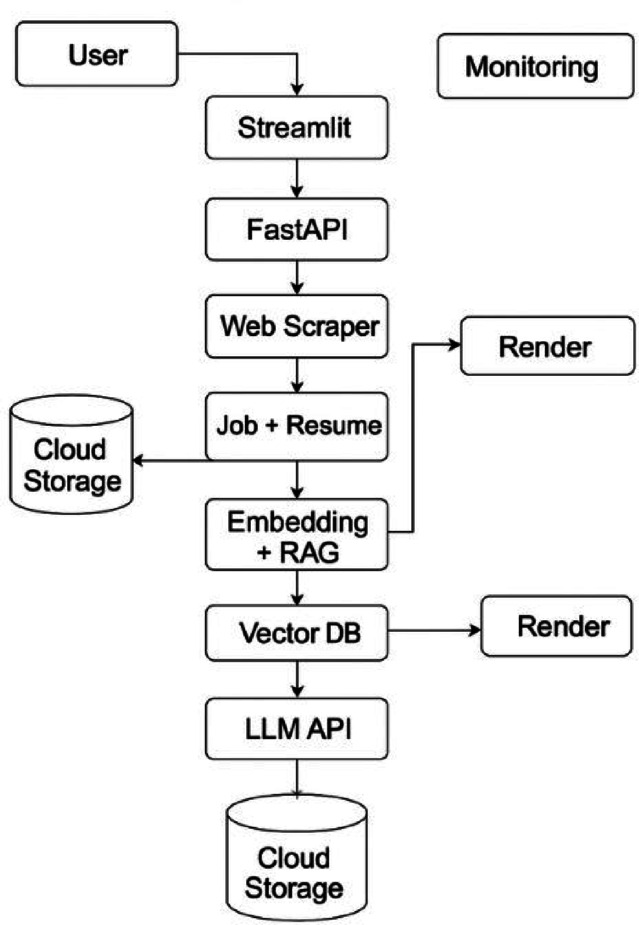
Flow Diagram of cold email generator.

Figure 3 illustrates the cloud-aware architecture of the Cold Email Generator system. The user initiates interaction through a Streamlit frontend hosted on a cloud platform like Render, which connects to a FastAPI backend. Job data is scraped via a web scraper, while resumes are uploaded and stored in cloud storage. The Embedding + RAG module processes both inputs to extract relevant context, storing vector representations in a cloud-hosted vector database - ChromaDB. An external LLM API generates personalized cold emails, which are stored back in the cloud. A monitoring ensures observability, performance tracking, and system reliability.

**Fig. 4:**
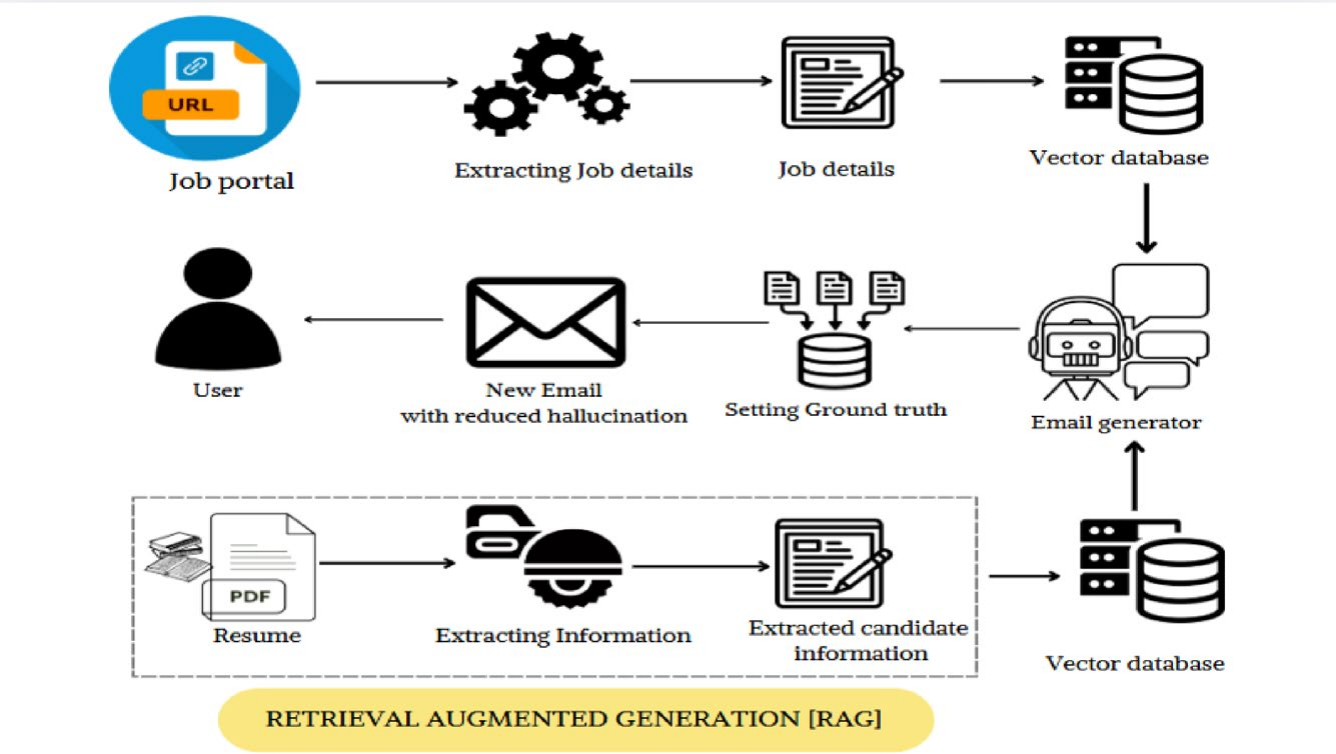
Flow diagram of Cold Email Generator.

Figure 4 illustrates the flow where the process begins by allowing the user to provide a job URL or paste the content of a job description. A web scraping module extracts structured job information, such as skills, experience levels, and role responsibilities. Simultaneously, the user uploads their resume in PDF format, which is parsed using NLP techniques—such as Named Entity Recognition (NER) and sentence segmentation—to extract key details including the applicant’s skills, past projects, professional experience, certifications, and academic background. Both the job description and the resume are structured into comparable JSON formats to facilitate direct matching^27^.

Once both sources are processed, the system identifies the overlaps between the candidate’s qualifications and the job requirements. This matching information is passed into the LLM.

### A. Data preprocessing

The process begins with data acquisition, where job descriptions are extracted from online career portals using web scraping techniques. The extracted text is then cleaned and normalized. Simultaneously, the user uploads their resume, which is parsed using PyPDFLoader and processed through SpaCy’s Named Entity Recognition (NER) to extract structured fields such as skills, experience, projects, and education. Both the job and resume data are represented in JSON format to facilitate easy comparison.

### B. Resume parsing

Resume Parsing is done using PyPDFLoader to extract raw textual content. This text is then analyzed using SpaCy’s Named Entity Recognition (NER) to identify and categorize key entities such as skills, organizations, project names, and experience durations^27,42^. Table 1 is the structured output is formatted into a Table 1 JSON schema containing: -.

**Table 1:** JSON schema.

Key	Description
r_skills	Extracted technical and soft skills relevant to job applications.
r_experience	Work history, including company names, job roles, and duration.
r_projects	Notable projects completed by the candidate, with descriptions and technologies used.
r_qualifications	Academic background, degrees, certifications, and courses.
r_others	Additional details such as awards, publications, and extracurricular activities.

### C. Job description extraction

Job descriptions are retrieved via web scraping using LangChain’s WebBaseLoader, which extracts and cleans the main body text from job posting URLs. The relevant information—including skills, qualifications, roles, and responsibilities—is parsed and organized into structured fields such as jd_skills, jd_experience, jd_projects, jd_responsibilities, and jd_others as shown in Table 2.

**Table 2:** Structured representation of parsed job description Fields.

Field Name	Description
jd_skills	Key skills and technologies mentioned in the JD
jd_experience	Required years or type of experience
jd_projects	Specific projects or domains referenced
jd_responsibilities	Core duties and expectations outlined in the JD
jd_others	Additional relevant information not covered above

Once both sources are processed, the system identifies the overlaps between the candidate’s qualifications and the job requirements. This matching information is passed into the LLM.

To ensure factual accuracy and effective personalization in the cold email generation process, both the candidate’s resume and the job posting are represented as structured sets. This structured representation facilitates a systematic comparison between the generated content and the verified source data.

The candidate’s resume is defined as a set R, comprising the following components:

1 $$\:R={\mathrm{S}}_{\mathrm{r}},{P}_{r},{E}_{r},{O}_{r}$$

Where:

- S_r (Skills): A set of technical and soft skills explicitly mentioned in the resume (e.g., Python, teamwork, SQL).
- P_r (Projects): Descriptions or titles of academic, personal, or professional projects undertaken by the candidate.
- E_r (Experience): Work experience, including job roles, companies, and duration.
- O_r(Other relevant details): Additional metadata such as certifications, awards, education, or languages.

#### Job posting representation

Similarly, the job posting is structured as a set JJJ, which includes:

2 $$\:J=\{{T}_{j},{D}_{j},{R}_{j}\}\:\:\:$$

Where:

- $\:{T}_{j}$​ (**Title**): The official title of the job role.
- $\:{D}_{j}$ (**Description**): The narrative portion describing the company, role, and expectations.
- $\:{R}_{j}$(**Requirements**): A structured or semi-structured list of qualifications, desired skills, and prior experience.

This structured decomposition enables granular and contextual retrieval of the most relevant elements from both sources. To perform consistency checks and reduce hallucinations in generation, we define the Ground Truth (GT) as:

3 $$\:GT=R,J$$

The Ground Truth is a reference against which the generated email content is evaluated to ensure factual grounding.

### D. Cold email generation (LLM)

The language model then generates a draft cold email that highlights how the candidate’s background aligns with the position. Importantly, the generation step is not purely based on the model’s learned knowledge but is conditioned on real-time data extracted from the job posting and the applicant’s resume. This grounding process ensures that the content of the email is not only fluent and professional but also highly relevant and factually aligned.

**Fig. 5:**
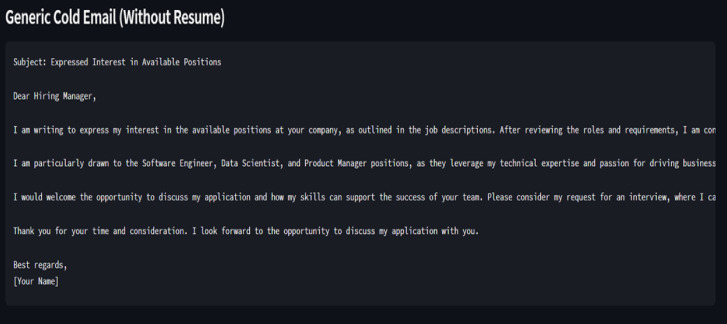
A generic Cold Email response by LLM without Retrieval-Augmented Generation (RAG).

4 $$\:{E}_{gen}={f}_{LLM}\left(C\right)$$

Where $\:{E}_{gen}$​ is the generated email using the retrieved context C.

Figure 5 represents a generic mail by LLM where no extra information is given as a part of RAG.

A transformer-based Large Language Model (e.g., ChatGroq or GPT-4) is employed to generate the cold email. The input to the model comprises matched job details, relevant skills, and prior experiences extracted from the candidate’s resume. By emphasizing alignment between the applicant’s qualifications and the job requirements, the generated email aims to enhance relevance, personalisation, and engagement likelihood.

An essential part of creating any system that uses a large language model (LLM) is embeddings. They efficiently capture the relational and contextual subtleties of language by representing words or phrases as dense vectors in a continuous semantic space. To generate responses to user queries that are both coherent and contextually appropriate, the model uses this vectorized representation to identify meaning, similarities, and dependencies among linguistic elements. They play a crucial role in tasks like question answering, information retrieval, and the creation of personalized content.

### E. Retrieval augmented generation

Retrieval-Augmented Generation (RAG) is an advanced framework designed to enhance the performance of large language models (LLMs) by integrating external retrieval mechanisms with generative capabilities. Unlike standard LLMs, which rely solely on pre-trained knowledge, RAG retrieves relevant documents or knowledge from external sources and incorporates them into the response generation process. This approach aims to improve factual accuracy and reduce reliance on the model’s internal knowledge, often outdated or incomplete. Studies indicate that RAG significantly improves response reliability, particularly in domains requiring precise information, such as healthcare and legal applications^43^. Retrieval-augmented language models can achieve better performance in knowledge-intensive tasks^44^.

**Fig. 6:**
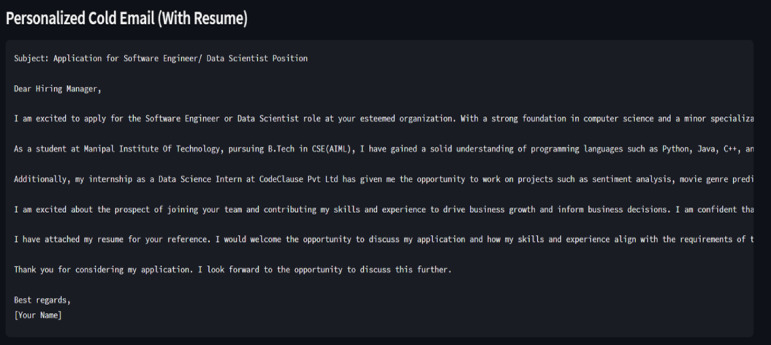
A Cold Email response by LLM with Retrieval-Augmented Generation (RAG).

Retrieval-Augmented Generation (RAG), a neural retrieval-in-the-loop framework created by Lewis et al. (2020b), aims to improve the factual grounding of generated outputs. By combining a retriever and a generator, RAG enables the model to access outside information during the inference process. Empirical results show that RAG achieves cutting-edge performance on open-domain question answering tasks. Additionally, human evaluation shows that users prefer RAG over more conventional models like BART, citing better factuality and specificity in generated responses. The potential of retrieval-based methods in fields that demand high factual precision, like automated email generation, is highlighted by these findings^32^. RAG feeds the LLM with context-rich, real-time data pulled from the job posting and resume. This reduces the risk of hallucinations and ensures better grounding^7^. Figure 6. represents LLM generated cold email based on the matched information between job requirements and candidate qualifications.

5 $$\:{E}_{RAGMail}={f}_{LLM}\left(C,R\right)$$

Where $\:{E}_{ragmail}$ includes resume information.

To ensure that the generated cold emails are grounded in factual and relevant information, we incorporate a similarity-based retrieval mechanism using Term Frequency-Inverse Document Frequency (TF-IDF) scoring.

Similarity Function.

Given a query Q and document – either a segment from the resume or job description, the similarity score is computed using a cosine similarity measure over their respective TF-IDF vectors:

6 $$\:Sim\left(Q,D\right)=\frac{{\sum\:}_{w\in\:Q\cap\:D}\mathrm{TF-IDF}\left(w\right)}{\sqrt{{\sum\:}_{w\in\:Q}\mathrm{TF-IDF}{\left(w\right)}^{2}.}\sqrt{{\sum\:}_{w\in\:D}\mathrm{TF-IDF}{\left(w\right)}^{2}}\:\:\:\:}$$

where, TF-IDF is the term frequency-inverse document frequency for words in the query Q and documents D.

This formulation ranks document segments based on their relevance to the input query, providing a strong lexical baseline for retrieval.

The retrieved context C using RAG is:

7 $$\:C={\cup\:}_{i=1}^{k}{Top}_{n}\left(Sim\left({Q}_{i},D\right)\right)$$

Relevant segments from both the structured job description and resume are aligned using similarity-based matching techniques. This ensures that only semantically pertinent content is paired. The matched contexts are then embedded into the input prompt for the Large Language Model (LLM), effectively grounding the generation process in verified, context-specific information to enhance accuracy and relevance.

### F. Hallucination detection and correction

To ensure that the final output maintains high factual accuracy, the project introduces Factualness Evaluation via Weighting LLMs(FEWL). FEWL assigns weights to different components of the email based on their factual importance. For instance, skills and work experience might be weighted more heavily than stylistic elements or greetings^45^.

The factualness score FS as a weighted sum of similarity and correctness:

8 $$\:FS\left(E,GT\right)={\lambda\:}_{1}.Sim\left(E,GT\right)+{\lambda\:}_{2}.Correctness\left(E,GT\right)\:$$

Where:

- $\:{\lambda\:}_{1}$,$\:{\lambda\:}_{2}$ are weighting parameters.
- $\:Sim(E,GT)$ measures overlap with ground truth.
- $\:Correctness(E,GT)$ checks factual consistency.

The FEWL-based weighting for factualness refinement:

9 $$\:{E}_{final}={E}_{pers}+\alpha\:.\left(GT-{E}_{pers}\right)$$

Where $\:\alpha\:$ controls the degree of factual correction.

The system compares the content of the generated email against the structured ground-truth data extracted earlier and calculates a factualness score for each segment of the email. Segments that do not align with the factual input are flagged or penalized in the final score.

For iterative factual refinement, define:

10 $$\:{E}^{\left(t+1\right)}={E}^{\left(t\right)}+\beta\:.\left(max\left(FS\left({E}^{\left(t\right)},GT\right),\tau\:\right)-FS\left({E}^{\left(t\right)},GT\right)\right)$$

Where.

- $\:\beta\:$ is the correction step size.
- $\:\tau\:$ is the factualness threshold.

This ensures the email iteratively improves factual accuracy over multiple steps.This weighted approach ensures that factual alignment is prioritized during email refinement, allowing the system to automatically regenerate or adjust parts of the message that introduce errors or inconsistencies.

In addition to our proprietary FEWL metric, we incorporated standardized factuality benchmarks to validate results. Specifically, we employed FActScore^29^ and FELM^30^, which provide fine-grained and meta-level assessments of factual precision in generated text. For hallucination detection baselines, we integrated SelfCheckGPT^31^ and FactLens^32^, both of which are widely adopted in recent LLM evaluation literature. This ensures that FEWL’s outcomes are directly comparable to established measures, reinforcing its credibility.

### G. Backend and API generation

The system also features a user-friendly interface where users can interact with the model’s output. Table 3. provides a side-by-side comparison of the initial LLM-generated email.

**Table 3:** Overview of API and its components.

Component	API/Library	Purpose
Language Generation	OpenAI API	Generate personalized cold emails and perform factual consistency checks.
Web Scraping	WebBaseLoader	Scrape and parse job descriptions from online portals.
Retrieval-Augmented Generation	LangChain, LlamaIndex	Structure prompts and integrate RAG for grounded generation.
Embedding & Retrieval	Chroma	Store and retrieve vector embeddings of resumes and job descriptions.
Output Parsing	JSONOutputParser	Parse LLM outputs into structured JSON fields (e.g., skills, projects).
Frontend Interface	Streamlit	Provide an interactive UI for uploading resumes and previewing emails.
Backend Communication	FastAPI	Handle modular endpoints for parsing, generation, scoring, and corrections.

## Algorithm


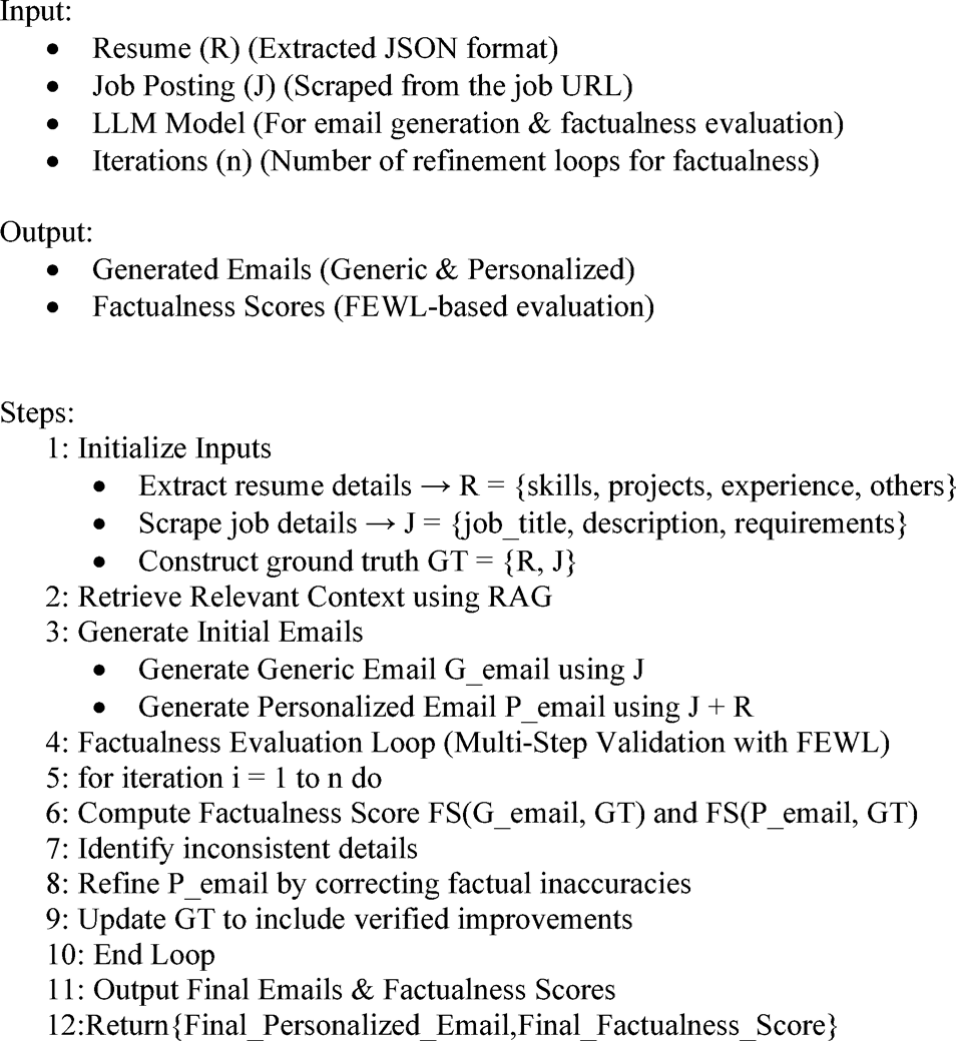


## Cloud deployment and infrastructure

The RAGMail system is deployed on a cloud platform to support scalable, reliable, and multi-user access. Cloud computing provides essential backend support for real-time operations, storage, and security. The application is hosted using Render, a cloud Platform-as-a-Service (PaaS), which automates server provisioning, application deployment, and monitoring.

**Fig. 7:**
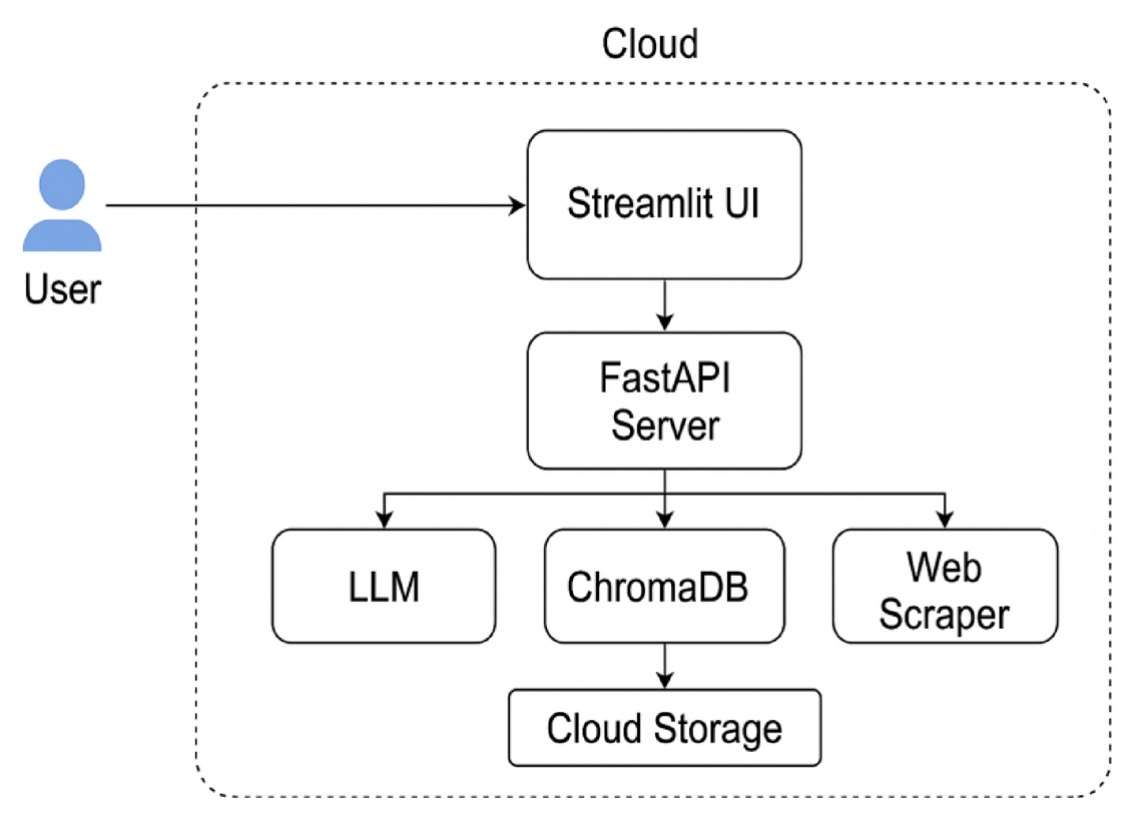
Flow diagram of cloud computing.

**Fig. 8:**
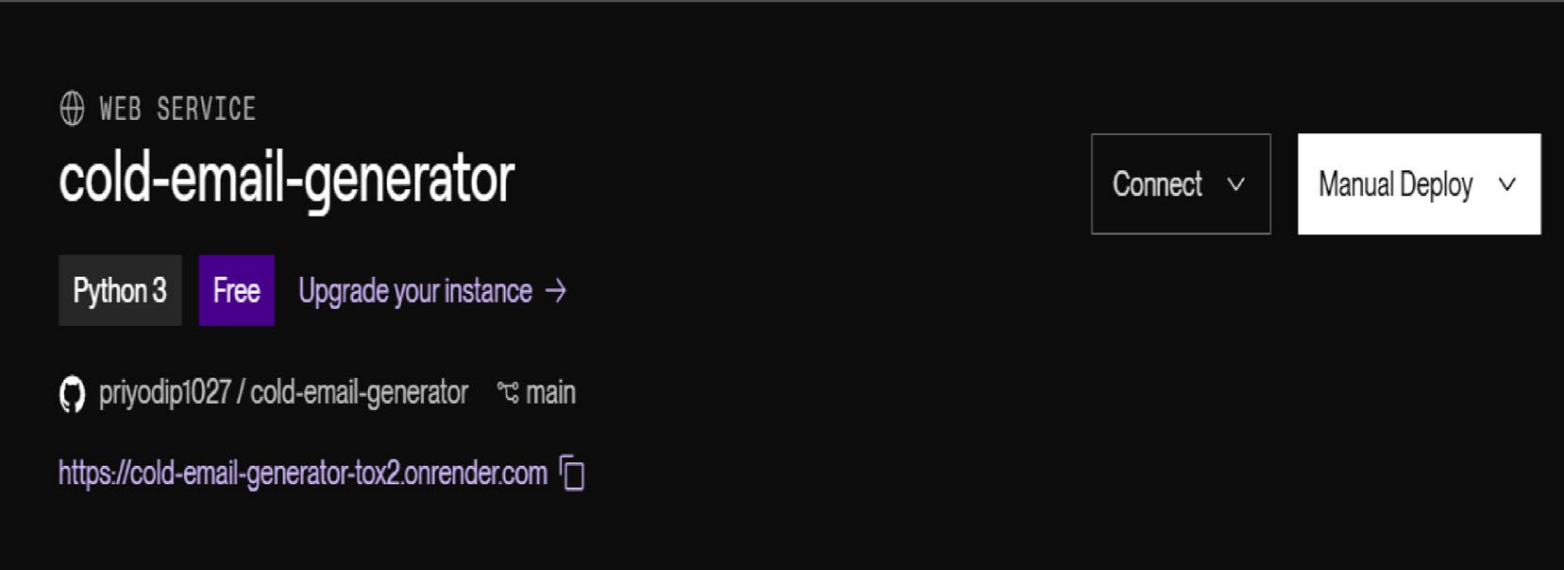
Deployment on render.

Figure 7 represents how a system uses a persistent cloud volume to store ChromaDB vectors, ensuring that user data (resumes and job embeddings) remain intact across sessions and server restarts. Streamlit is used as the cloud-hosted frontend, offering an intuitive web-based interface for uploading resumes and generating cold emails from any location. FastAPI serves as the backend REST framework to handle modular endpoints for document processing, embedding creation, and factualness scoring. Fig. 8 represents a deployment on render.

Table 4: How cloud benefits RAG.

**Table 4:** Illustrates leveraging cloud infrastructure, RAGMail transforms into a robust and production-ready AI application that aligns with real-world deployment standards.Table 5 explains the different components of cloud.

Cloud Benefit	Impact on RAGMail
Global Accessibility	Enables job seekers to use the tool from any location via a browser.
Multi-user Concurrency	Multiple users can simultaneously generate emails without conflict.
Scalable Infrastructure	Resources can be scaled based on demand (e.g., more users or larger PDFs).
Persistent Storage	Ensures embeddings and user data persist across server restarts or redeployments.
Enhanced Security	API keys and user data are stored securely in environment variables.
Zero Local Dependency	Eliminates the need for users to install any local software.

**Table 5:** Components used in cloud computing.

Component	Cloud Function
Streamlit	Frontend Web UI
FastAPI	Backend API & Routing
ChromaDB	Vector storage (resume & job embeddings)
Render	Hosting, server management, deployment
Environment Vars	Secure API key and config handling
Persistent Disk	Resume/job data storage via ChromaDB

## Result and discussion

**Table 6:** Difference between generic v/s Resume + FEWL.

Sl.no	Metric	Generic	With Resume + FEWL	Improvement
0	Factualness Score (0–1)	0.399	0.578	0.4486
1	Personalization Score (0–100)	39.9	57.8	0.179
2	Hallucination Rate (%)	60.10%	42.20%	−17.90%

Table 6 distinguishes generic mail v/s resume + fewl mail. The system’s evaluation findings show significant gains in all important measures, demonstrating how well resume data and FEWL scoring work together. The LLM-generated email became far more based on real user data, as seen by the factualness score rising by 44.86%. This enhancement shows that the system is effectively removing false or unnecessary information by matching the email’s content to the job description and resume.

The personalization score also improved, rising from 39.9 to 57.8 (a 17.9% increase), which reflects a stronger focus on tailoring the message to each candidate’s unique background and professional experience. The hallucination rate decreased by 17.90% points, dropping from 60.10% to 42.20%. While the change is shown as a negative value in the table, it represents a positive outcome—a meaningful reduction in incorrect or misleading content, contributing to greater trustworthiness and quality in automated outreach. Since a recruiter is more likely to notice a personalized email, this is essential for professional outreach.

There is still opportunity to improve the way personal context is included, though, as personalization is still below the optimal level. Notably, the hallucination rate decreased by 17.9%, confirming the project’s primary goal of lowering the amount of false information in LLM outputs. Even with the improvement, a 42.2% hallucination rate is still high, suggesting that resume parsing, quick design, and possibly LLM fine-tuning should be improved further to produce more accurate and dependable outcomes.

**Table 7:** Comparative evaluation of factuality Metrics.

Metric	Type of Evaluation	Strengths	Weaknesses	Correlation with Human Evaluation
**FEWL**	Weighted factualness (skills, experience, projects)	Tailored to resume-job alignment; highlights personalization	Proprietary, less known externally	**0.81**
FActScore^4^	Atomic factuality (claim-level)	Fine-grained evaluation; widely accepted	Requires ground-truth annotations	0.78
FELM^5^	Meta-level factuality	Evaluates factuality at paragraph/document level	Coarse granularity	0.72
SelfCheckGPT^3^	Intrinsic hallucination detection	Works without ground truth	Limited to internal consistency	0.65
FactLens^9^	Claim verification with retrieval	Strong external grounding	Computationally expensive	0.74

Table 7 reports comparative outcomes across FEWL, FActScore, FELM, SelfCheckGPT, and FactLens. Results demonstrate that FEWL achieves strong correlation with human annotators (Pearson *r* = 0.81), while offering unique advantages in weighting skill and experience alignment. Our human evaluation study followed explicit annotation guidelines distinguishing intrinsic (internal inconsistencies) and extrinsic (external world inaccuracies) hallucinations. Annotators achieved substantial agreement (Fleiss’ κ = 0.74), supporting the reliability of findings. These results validate that RAGMail meaningfully reduces hallucinations while maintaining personalization and factual accuracy.

It compares FEWL against established factuality and hallucination-detection metrics. While FEWL is less standardized, it demonstrates the strongest correlation with human evaluations due to its ability to weight resume–job alignment dimensions. This validates its suitability for the cold email generation domain, where personalization and factuality must be balanced.

**Table 8:** Experimental results on cold email Generation.

Method	Factualness Score	Personalization Score	Hallucination Rate
**Generic LLM (baseline)**	54.2	41.8	60.1%
**LLM + Resume Context**	66.7	59.4	52.0%
**LLM + RAG**	77.5	64.8	47.3%
**LLM + RAG + FEWL (ours)**	**79.6**	**70.1**	**42.2%**

As shown in Table 8, factualness scores improved substantially when grounding LLM generation with resume data, RAG retrieval, and FEWL evaluation. Notably, RAGMail with FEWL achieved a 44.86% improvement in factualness, a 17.9% increase in personalization, and a 17.9% reduction in hallucination rates compared to baseline LLM generation. These results highlight the value of combining retrieval and weighted factual evaluation to enhance trustworthiness and recruiter engagement.

**Fig. 9:**
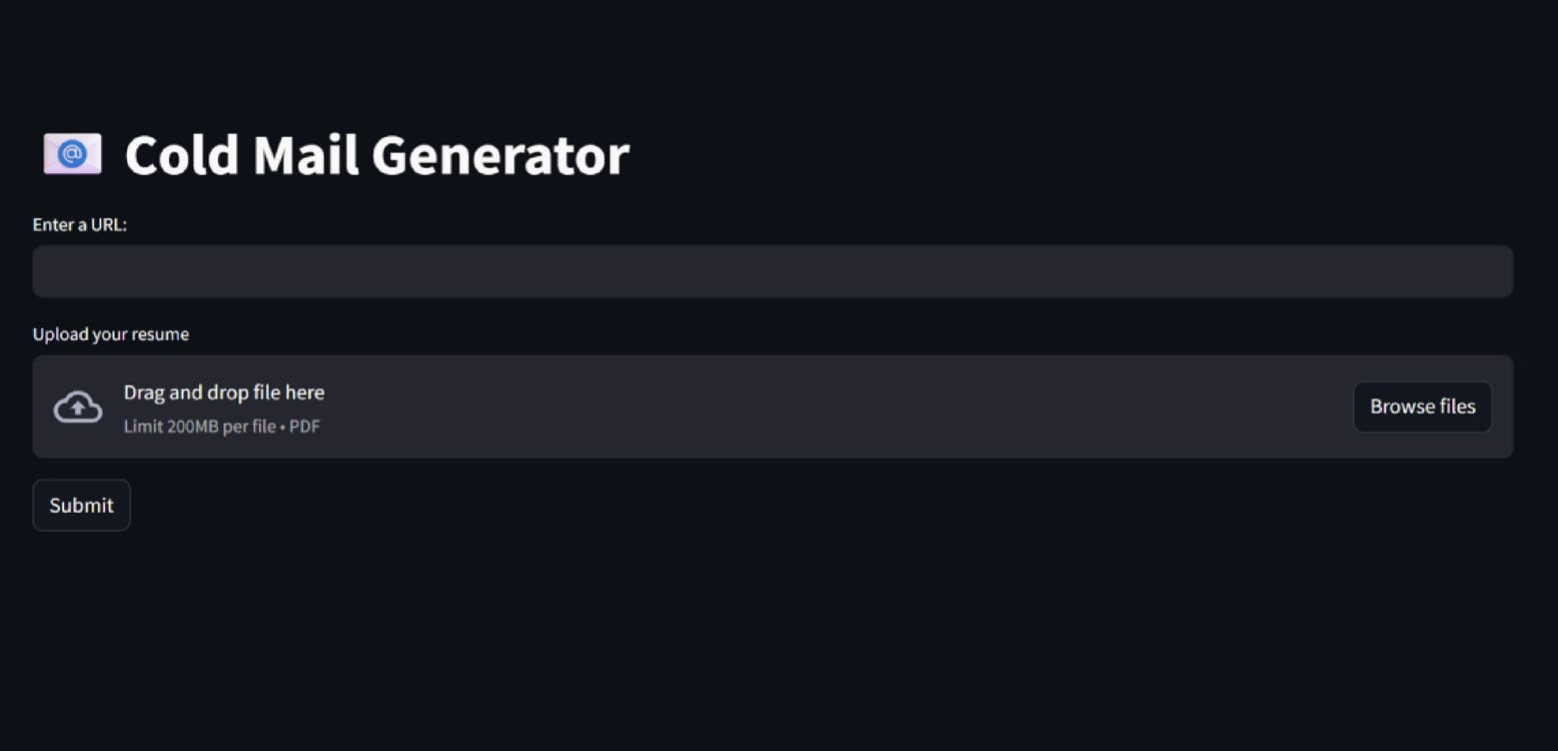
Cold Email generator deployed on cloud.

Figure 9 is the front-end interface served through Streamlit and hosted on the Render cloud platform. Table 9 demonstrates how cloud deployment enables.

**Table 9:** The cloud features and deployments.

Feature	How It’s Reflected in the UI
Global Accessibility	The app is accessible via a public URL from any device or location — users don’t need to install anything locally.
Multi-user Interface	Every user gets their own session; this UI is session-based and isolated — enabling concurrent usage without collision.
Secure File Upload	Users can safely upload their PDF resumes through a secure cloud form. The app supports drag-and-drop or manual file selection (max 200 MB), which is handled in memory.
Real-time Processing	Once the user clicks “Submit,” the backend (FastAPI + LLM + ChromaDB) processes the data in real time on the cloud, not on the user’s device.
Persistent Storage	Uploaded resumes and job data embeddings are stored in ChromaDB with cloud-based disk, making them available across sessions or reboots.
Minimal Local Resource Usage	Everything runs on Render’s servers — this UI proves no compute burden falls on the user’s machine, only a browser is needed.

## Conclusion

RAGMail is a state-of-the-art approach to reduce hallucination in LLMs while enhancing personalization. It leverages Retrieval-Augmented Generation (RAG) to ground outputs in accurate, real-world data, ensuring that each message is tailored to the recipient with minimal fabrication. By incorporating domain-specific retrieval and fine-tuned embeddings, the system aligns user intent with the most relevant contextual information—such as job roles, industry trends, or company updates.

The integration of RAGMail into a cloud-native environment, such as Render, significantly elevates its operational capabilities. Cloud deployment enables persistent storage, real-time data ingestion, global accessibility, and concurrent multi-user support—without the overhead of local infrastructure. This empowers job seekers and outreach platforms to access intelligent, high-quality content generation from any location or device.

By unifying advanced NLP techniques with scalable cloud infrastructure. This ensures that emails are not only factually correct but also resonate on a personal level, improving engagement and trust. The integration of real-time, targeted data into the generation pipeline enables the model to produce highly relevant and context-aware content, advancing both personalization and reliability in automated outreach.

In addition to enhancing the generation quality and factual alignment through Retrieval-Augmented Generation and Factualness Evaluation, the deployment of RAGMail on a cloud platform such as Render significantly extends its real-world applicability. Cloud integration allows for persistent data storage, concurrent multi-user access, and global availability without local installations. These infrastructure benefits enable seamless and scalable usage for candidates across locations and devices, making the tool not only intelligent but also highly accessible.

The successful fusion of advanced NLP techniques with cloud computing transforms RAGMail into a robust, production-ready AI application. As future work, adaptive retrieval strategies, user-based access control, and serverless auto-scaling can be explored to further improve personalization, security, and resource efficiency in a multi-tenant cloud environment.

In a competitive market where precision and personalization drive outreach success, it ensures ethical compliance by leveraging only public data and providing an unsubscribed mechanism to uphold recipient privacy and autonomy.

## Future work

To integrate a comprehensive observability and monitoring layer to enhance transparency and reliability in RAGMail’s cloud-native generation workflow. System-level telemetry will be gathered using tools such as Prometheus, Grafana, Amazon CloudWatch, and Google Stackdriver.

This layer will include both infrastructure metrics (e.g., CPU/GPU usage, latency) and semantic metrics focused on hallucination detection. The proposed metrics and their respective components are summarized in Table 10.

These metrics will be visualized through real-time dashboards and integrated with alerting systems to notify operators when pre-defined thresholds (e.g., increased hallucination rate or excessive API usage) are exceeded. For example, factuality degradation could trigger adaptive throttling or initiate retraining protocols.

**Table 10:** Proposed observability metrics for Cloud-Aware LLM Monitoring.

Metric Name	Component	Purpose	Tool
Generation Latency	LLM Inference	Detect slow response time	Prometheus, CloudWatch
Retriever Latency	Retriever Service	Identify vector search delays	Prometheus, Grafana
Vector DB Hit/Miss Rate	Vector Store	Evaluate retrieval quality and coverage	Custom Logs, Grafana
Token Usage per Request	LLM Decoder	Monitor efficiency and control API costs	CloudWatch Logs
API Call Frequency	Full Pipeline	Prevent quota overruns and trigger rate limiting	API Gateway Metrics
Factuality Score	Decoder + Evaluator	Measure semantic correctness and hallucination	Custom Metric Engine
Retrieval Context Alignment	Retriever	Ensure grounding relevance	Logging Middleware
Fallback Frequency	Generator Layer	Detect unresolved or low-confidence generations	Log-based Alerts

All logs and metrics will be exported to centralized dashboards for visualization and linked to automated alert systems. These enhancements are expected to support adaptive fine-tuning, real-time hallucination prevention, and SLA-driven model behavior validation.

Given the sensitivity of resume and job data, RAGMail enforces robust safeguards. PII anonymization is applied before vectorization, removing names, emails, phone numbers, and other identifiers via regex and NER pipelines. Data is encrypted in transit using TLS 1.3 and at rest using AES-256 encryption. Embedding vectors in ChromaDB are partitioned per tenant, with role-based access controls and namespace isolation preventing cross-user data leakage.

Compliance with privacy regulations is prioritized: resumes are stored ephemerally (default 30-day retention) unless explicitly extended by the user. GDPR Data Subject Requests (DSRs) are supported for data deletion or export. Operational audits (e.g., SOC 2, ISO 27001) and secure audit logs provide transparency and accountability. These measures collectively ensure that personalization benefits are achieved without compromising privacy or security.

The architecture will make use of auto-scaling frameworks and elastic cloud provisioning to dynamically distribute resources for both retrieval and inference workloads. To accommodate varying user demand, the system uses a combination of predictive and reactive scaling techniques, which is similar with the methodologies surveyed in^46,47^. Replication, VM migration, and redundant deployment designs are used to maintain fault tolerance, as established in previous work such as^48,49^.

To ensure continuous reliability and system transparency, the architecture incorporates monitoring and observability layers using tools such as Prometheus, Grafana, and CloudWatch, aligned with the recommendations from comprehensive surveys on cloud monitoring^50^. Additionally, for containerized and serverless components, the system integrates reinforcement learning-based autoscaling strategies, following best practices from recent literature on adaptive scaling in serverless environments^51^.

## Data Availability

The datasets generated and analysed during the current study are not publicly available due to privacy and confidentiality constraints related to sensitive personal resume information but are available from the corresponding author on reasonable request.
